# Restless Leg Syndrome and Sleep Quality in Lumbar Radiculopathy Patients

**DOI:** 10.1155/2014/245358

**Published:** 2014-07-08

**Authors:** Ersoy Kocabicak, Murat Terzi, Kursad Akpinar, Kemal Paksoy, Ibrahim Cebeci, Omer Iyigun

**Affiliations:** ^1^Department of Neurosurgery, Ondokuz Mayis University, Faculty of Medicine, 55200 Samsun, Turkey; ^2^Departments of Neurosurgery and Neuroscience, Maastricht University Medical Center, P. Debyelaan 25, 6202 AZ Maastricht, The Netherlands; ^3^Department of Neurology, Ondokuz Mayis University, Faculty of Medicine, 55200 Samsun, Turkey

## Abstract

*Background*. To investigate the frequency of restless leg syndrome (RLS), sleep quality impairment, depression, fatigue, and sleep behavior disorder and to determine the effects of surgery on these parameters in radiculopathy patients resistant to conservative treatment. *Methods*. The present study included 66 lumbar radiculopathy patients, who were resistant to conservative treatment and had indication of surgery. Five different questionnaires were performed to assess depression (the Beck Depression Inventory (BDI)), sleep quality (the Pittsburgh Sleep Quality Index (PSQI)), fatigue (the Fatigue Severity Scale (FSS)), and presence of RLS and rapid eye movement sleep behavior disorder (RBD). The same questionnaires were also performed on a control group (*n* = 61). *Results*. Of the radiculopathy patients, 68.1% had RLS and 92.4% had fatigue. Of the controls, 16.4% had RLS and 59% had fatigue. RBD was present in 8 (12.1%) patients and 3 (4.9%) controls. The PSQI revealed that sleep quality was impaired in 46 (69.7%) patients and 35 (57.4%) controls (*P* > 0.05). The number of individuals having substantial depression according to the BDI was significantly higher in the patients than in the controls. *Conclusions*. There was a significant increase in the frequency of RLS, which was significantly decreased in the postoperative period in the radiculopathy patients.

## 1. Introduction

Radiculopathies occur due to root compression secondary to ligament hypertrophy accompanying disc herniation and protrusion and/or disc degeneration frequently in cervical and lumbosacral regions [1, 2]. Radiculopathy is observed in 50% of humans on average and is one of the most important causes of morbidity that leads to substantial restrictions in social life by causing serious medical and socioeconomic problems [3, 4]. The most common discopathies are L5-S1 and L4-L5 discopathies, followed by L3-L4 and L2-L3 discopathies that are defined as high disc discopathies [5, 6]. Restless leg syndrome (RLS) is a sensorimotor disorder including discomfort in lower limbs, which is usually worsened in the evening, relieved with motion, and worsened at rest [7]. Rapid eye movement (REM) sleep behavior disorder (RBD) is a parasomnia associated with destructive and detrimental behaviors during the REM period of sleep and increased tonus in the extremities on electromyography [8]. Although radiculopathy is one of the secondary risk factors for RLS, to the best of our knowledge, there is no study investigating the presence of RLS and a potential RBD in patients diagnosed with radiculopathy.

The present study aimed to investigate the frequency of RLS, sleep quality impairment, depression, fatigue, and RBD, which are likely to affect quality of life, and to determine the effects of surgery on these parameters in patients with radiculopathy resistant to conservative treatment.

## 2. Materials and Methods

The present study included 66 patients diagnosed with lumbar radiculopathy, who were resistant to conservative treatment and had indication of surgery for lumbar disc herniation between May 2012 and November 2012 in the Department of Neurosurgery and Neurology of Ondokuz Mayıs University, Faculty of Medicine. Five different questionnaires were performed on the patients to assess depression (the Beck Depression Inventory (BDI)), sleep quality (the Pittsburgh Sleep Quality Index (PSQI)), fatigue (the Fatigue Severity Scale (FSS)), and the presence of RLS and RBD [7, 8]. The same questionnaires were also performed on a control group comprising 61 healthy subjects. Control group was formed from volunteers that were admitted to the neurology clinic during the study period and did not have any known risk factors for RLS. Patients with a history of polyneuropathy, Parkinson's disease, poliomyelitis, Isaacs' syndrome, hyperplexia, diabetes mellitus, uremia, gastrectomy, malignancy, peripheral vascular disease, and rheumatoid arthritis, which are among the etiology of RLS and familial RLS, and those who were pregnant were excluded. Patients receiving medical treatment for lumbar radiculopathy for at least six weeks were included in the study. Patients with spinal stenosis and acute radicular pain or acute leg weakness were excluded. Patients who underwent a previous spinal surgery and those who received caffeine, calcium channel blockers, lithium, neuroleptics, alcohol, and sedatives were also excluded. Ethical approval was obtained from the Medical Research Ethics Committee. Written and signed informed consent was obtained from all patients and controls.

Surgical intervention was decided based on examination and clinical findings and results of magnetic resonance imaging of the patients. The questionnaires were performed on the patients within one week prior to the surgery and one month after the surgery. RLS was diagnosed based on the RLS diagnostic criteria of the International Restless Legs Syndrome Study Group (IRLSSG), and RBD was diagnosed based on the clinical criteria defined in the International Classification of Sleep Disorders (ICSD) [8]. In addition to the association of extremity or body movements with dream state, the presence of at least one of the criteria including harmful sleep behaviors, the patient's living the dream state, or interruption of sleep due to sleep behaviors was considered as minimal diagnostic criteria and patients meeting these criteria were clinically diagnosed with RBD. The Stavanger Sleep Questionnaire was performed on the patients diagnosed with RBD and on the subjects in the control group. All questionnaires were performed by the same person via face-to-face interview. Those having a score of 27 or higher from the FSS, a score of 5 or higher from the PSQI, and a score of 9 or higher from the BDI were considered abnormal.

All questionnaires were performed within one week during the preoperative period and at the first postoperative month. Symptoms were considered as pain, paresthesia, hypoesthesia, and leg weakness under the heading of radiculopathy. However, effects of dominant complaint and symptom duration during preoperative period on postoperative RLS have not been evaluated. We evaluated all symptoms under the heading of radiculopathy. Improvement in quality of life of patients might have started prior to one month postoperatively. We waited for a period of one month during postoperative period and aimed to determine the long-term effect of surgery applied on clinical picture.

Statistical analyses were performed using the Statistical Package for the Social Sciences (SPSS, Inc., Chicago, IL, USA) version 15.0. Continuous data were expressed as mean ± standard deviation, whereas frequency data were expressed in percentages (%). Intergroup comparisons were performed by independent sample *t*-test for continuous data and by chi-square test for categorical data. Intragroup and intergroup comparisons for RLS, fatigue, depression, RBD, and sleep quality impairment were carried out separately. A *P* value <0.05 was considered statistically significant.

## 3. Results

The present study included 66 patients diagnosed with lumbar radiculopathy and resistant to conservative treatment and 61 healthy subjects. The general characteristics of the patient group are presented in Table 1.

The control group comprised 35 females and 26 males (57.4%/42.6%) and the patient group comprised 40 females and 26 males (60.6%/39.4%). The mean age of the patient and control groups was 39.6 ± 4.3 years and 41.1 ± 6.4 years, respectively. There were no significant differences between the patient and control groups in terms of age and gender (*P* > 0.05). Of the radiculopathy patients, 68.1% had RLS and 92.4% had fatigue. Of the control group, 16.4% had RLS and 59% had fatigue. RBD was present in 8 (12.1%) of the patients and 3 (4.9%) of the controls. The PSQI revealed that sleep quality was impaired in 46 (69.7%) of the patients and in 35 (57.4%) of the controls. The number of individuals having substantial depression according to the BDI was significantly higher in the patient group as compared to the control group (Table 2).

In the patients with radiculopathy, the hernia being on the right or left side and the hernia being unilateral or bilateral, as well as the type and level of hernia, did not significantly affect the variables. When the patients with lumbar radiculopathy were reevaluated by the quality of life questionnaires in the postoperative first month, all patients having RBD in the preoperative period still had RBD in the postoperative period. Nevertheless, significant decreases in fatigue and depression, as well as in the frequency of RLS, were observed in the patient group in the postoperative period; improvement in the sleep quality was also observed (Table 3).

## 4. Discussion

The most common causes of the lumbar radiculopathy are herniation of the lumbar intervertebral disc into the spinal canal or neural foramen or degenerative lumbar spinal stenosis [9]. Alterations in related reflexes and presence of radicular symptoms (pain, paresthesia, and weakness) may occur due to nerve root compression [10, 11]. Diseases leading to peripheral nerve injury such as chronic radiculopathy may cause neuropathic pain [12, 13]. The number of studies investigating the quality of life in patients diagnosed with radiculopathy is quite limited. Bosković et al. used the Short Form 36 Health Survey (SF-36) questionnaire to assess quality of life of patients with lumbar radiculopathy and demonstrated an increase in physical health scores with conservative or surgical treatment, which were low [10, 11, 14]. Likewise, Albert et al. showed an improvement in the quality of life of patients with lumbar radiculopathy after surgery [15]. Unlike other studies, the present study widely evaluated the quality of life of patients with lumbar radiculopathy in terms of five different parameters both before and after the surgery and compared the results with those of the control group.

The frequency of RLS was demonstrated to be 3%–10% in the general population [16]. RLS may be idiopathic (primary) or secondary [17]. Symptomatic RLS in old people has been considered secondary to other diseases such as uremia, diabetes mellitus, anemia, peripheral polyneuropathy, gastric surgery, Parkinson's disease, and rheumatoid arthritis [2, 18]. In the present study, RLS is accompanied in 68.1% of the patients with lumbar radiculopathy in the preoperative period and there was a significant improvement in the postoperative first month. The presence of radiculopathy can be considered as a very strong independent risk factor for RLS due to the fact that other risk factors such as uremia and diabetes likely to cause RLS were absent in our patients and a significant improvement was observed in the postoperative period. Primary criteria for diagnosis of RLS are considered as unpleasant feelings or disturbance in extremities, particularly in legs, together with urge to move, starting or worsening of unpleasant feelings, or urge to move during immobile situations such as rest or sitting, partial or complete improvement in these conditions with movements such as walking, rubbing, and swinging for at least as long as the activity is continued, worsening of symptoms at the evening or night compared to daytime, or occurrence of symptoms at evening/night. However, positive family history, response to dopaminergic therapy, periodical limb movements, normal neurological examination, sleep disturbances, and presence of associated symptoms are supportive criteria for diagnosis. These criteria have been taken into account for all patients included in the study. Manifestations such as cramps, paresthesia and sensory symptoms, and positional comfort disorder have been excluded. By considering all of these criteria, frequency of RLS has been found to be higher in patient group compared to control group and postoperative period in our study. Frequency of RLS has been found to be 16.4% in control group, slightly higher than that observed in other investigations.

Etiopathogenesis of RLS has not been elucidated yet. The great majority of patients benefiting from dopaminergic drugs and iron therapy, as well as low ferritin and high transferrin levels in cerebrospinal fluid of RLS patients, support the hypothesis that low levels of brain iron lead to dopaminergic dysfunction and thus symptoms are developed [19]. There is a strong relation between iron deficiency and dopamine deficiency [20]. We are in the opinion that not measuring blood iron levels of the patients is the limitation of the present study.

Restless leg syndrome is diagnosed by clinical inquiry and detailed anamnesis. Walters et al. (1995) conducted a study on behalf of the IRLSSG in 1995 and published the diagnostic criteria for the disease; these criteria were reviewed in 2002 by the IRLSSG and a rating scale for the severity of RLS was published together with diagnostic criteria of RLS in 2003 [7, 21]. In order to diagnose the disease, an individual must have all of the four main criteria in this scale. Although radiculopathy can be a secondary risk factor for RLS, to our knowledge, there is no study investigating the frequency of RLS in radiculopathy patients in the literature [21]. Although the origin of radicular pain is unclear, animal and human studies have demonstrated a decrease in root blood flow due to compression on dorsal root ganglion and radix [22, 23]. There are studies reporting that cytokines such as interleukin- (IL-) 6 and IL-1 and tumor necrosis factor- (TNF-) alpha are increased in the cerebrospinal fluid of radiculopathy patients due to this compression and these agents cause radicular pain [24, 25]. Pain and other clinical signs that appear along with peripheral nerve involvement in radiculopathy patients may affect RLS-associated anatomic configurations in the CNS and may cause alterations in the levels of neurotransmitters such as dopamine. As a result, clinical manifestations of RLS may occur. Unclear relation of cytokines with dopamine may play a role in this association. Pain may be a factor triggering RLS in patients with lumbosacral radiculopathy and possible decrease in pain during postoperative period may be associated with decrease in frequency of RLS. We did not investigate the pain in our study. Studies investigating the effects of pain in patients with lumbosacral radiculopathy on quality of life and RLS may be conducted. The patients did not have a history of any kind of drug intake such as antidepressant or sedative drugs in both the preoperative period and the first month postoperatively. Simple analgesics were occasionally used for pain in the preoperative and postoperative periods. Medical treatment in the postoperative period was not different than that in the preoperative period. As another issue, the frequencies of RLS and RBD in the control group were slightly higher than normal population studies. Between patient and control groups did not difference in terms of age and gender. Also, we applied the same questionnaire to both groups. Fatigue is another parameter that unfavorably influences daily life activities. Fatigue was present in 92.4% of our patients in the preoperative period and a significant improvement was observed in the postoperative period. Fatigue can be frequently encountered in the CNS diseases; however, its association with peripheral nervous system diseases is not well known. Not enjoying life, reluctance, pain, and depression, which may be observed in radiculopathy patients particularly in the event of unresponsiveness to conservative treatment, may increase fatigue [10, 11]. In the present study, we also observed depression to be more common in the patients with radiculopathy. Depression may occur as a reaction of the patient against disease itself and unresponsiveness to conservative treatments. An underlying common pathophysiological mechanism may be responsible for the comorbidity of RLS, fatigue, and depression.

The patients have live dreams that are accompanied by extremity movements, such as loss of muscle atonia, kicking, punching, and puncturing, which are not supposed to be normally present during the REM sleep, and this usually results in injury to the partner in bed [26]. The frequency of RBD in the general population ranges from 0.38% to 0.5% [27, 28]. In the present study, patients who were diagnosed with RBD based on the minimal diagnostic criteria of RBD defined in ICSD were evaluated. Polysomnography was not performed on the patients with clinical RBD. Thus, the clinical diagnosis of some of the patients with RBD might be false positive. To our knowledge, studies investigating the frequency of RBD in radiculopathy patients are lacking; therefore, further large-scale studies are needed on this issue. To the best of our knowledge, the present study is the first study in the literature investigating the presence of RLS and RBD in patients diagnosed with radiculopathy.

## 5. Conclusions

Radiculopathy may be accompanied by RLS and RBD and this coexistence might be important in terms of its negative impacts on the quality of life of patients. RLS and RBD in radiculopathy patients may be associated with both the direct impact of the disease itself and the existing depression and fatigue. The results of the present study revealed that there was a significant increase in the frequency of RLS and that this frequency was significantly decreased in the postoperative period in the patients with radiculopathy. Further large-scale studies with longer follow-up periods, which would additionally investigate the association between cytokines and dopamine, are required on this issue.

## Figures and Tables

**Table 1:** General characteristics of the patients with lumbar radiculopathy.

Characteristics	Patient group (*n* = 66)
Age (mean ± SD), years	39.6 ± 4.3
Gender, *n* (%)	
Male	26 (39.4)
Female	40 (61.6)
Level of disc hernia, *n* (%)	
L2-L3	5 (0.75)
L3-L4	11 (16.6)
L4-L5	28 (42.4)
L5-S1	22 (33.3)
Dominant complaint, *n* (%)	
Radiculopathy	66 (100)

Leg weakness	21 (31.8)

**Table 2:** Comparison between the patients with lumbar radiculopathy and controls in terms of quality of life parameters in the preoperative period.

Parameters	Control group (*n* = 61) *n* (%)	Patient group (*n* = 66) *n* (%)	*P*
Fatigue	36 (59)	59 (92.4)	**<0.001**
Presence of RBD	3 (4.9)	8 (12.1)	**0.001**
PSQI	35 (57.4)	46 (69.7)	**>0.05**
Depression	25 (41)	62 (93.9)	**<0.001**
Presence of RLS	10 (16.4)	45 (68.1)	**<0.001**

RBD: REM sleep behavior disorder, PSQI: Pittsburg Sleep Quality Index, and RLS: restless leg syndrome.

**Table 3:** Comparison between preoperative and postoperative quality of life parameters in the patients with lumbar radiculopathy.

Parameters	Preoperative period *n* (%)	Postoperative period *n* (%)	*P*
Fatigue	59 (92.4)	6 (9.1)	**<0.001**
Presence of RBD	8 (12.1)	8 (12.1)	**>0.05**
PSQI	46 (69.7)	30 (45.5)	**0.005**
Depression	62 (93.9)	8 (12.1)	**<0.001**
Presence of RLS	45 (68.1)	16 (24.2)	**<0.001**

RBD: REM sleep behavior disorder, PSQI: Pittsburg Sleep Quality Index, and RLS: restless leg syndrome.
